# The development of theory-informed participant-centred interventions to maximise participant retention in randomised controlled trials

**DOI:** 10.1186/s13063-022-06218-8

**Published:** 2022-04-08

**Authors:** Rumana Newlands, Eilidh Duncan, Shaun Treweek, Jim Elliott, Justin Presseau, Peter Bower, Graeme MacLennan, Margaret Ogden, Mary Wells, Miles D. Witham, Bridget Young, Katie Gillies

**Affiliations:** 1 Health Services Research Unit, Health Sciences Building, Foresterhill, Aberdeen, UK; 2 Public Partner, Aberdeen, UK; 3 Clinical Epidemiology Program, Ottawa Hospital Research Institute, Ottawa, Canada; 4 School of Epidemiology and Public Health, University of Ottawa, Ottawa, Canada; 5 School of Psychology, University of Ottawa, Ottawa, Canada; 6 NIHR School for Primary Care Research, Centre for Primary Care and Health Services Research, Manchester Academic Health Science Centre, University of Manchester, Manchester, UK; 7 Faculty of Medicine, Department of Surgery and Cancer, Imperial College, London, UK; 8 Imperial College Healthcare NHS Trust, London, UK; 9 AGE Research Group, NIHR Newcastle Biomedical Research Centre, Newcastle University and Newcastle upon Tyne Hospitals NHS Foundation Trust, Newcastle, UK; 10 Department of Public Health, Policy and Systems, Institute of Population Health, University of Liverpool, Liverpool, UK

**Keywords:** Retention, Clinical trials, Intervention development, Theory, Behaviour

## Abstract

**Background:**

A failure of clinical trials to retain participants can influence the trial findings and significantly impact the potential of the trial to influence clinical practice. Retention of participants involves people, often the trial participants themselves, performing a behaviour (e.g. returning a questionnaire or attending a follow-up clinic as part of the research). Most existing interventions that aim to improve the retention of trial participants fail to describe any theoretical basis for the potential effect (on behaviour) and also whether there was any patient and/or participant input during development. The aim of this study was to address these two problems by developing theory- informed, participant-centred, interventions to improve trial retention.

**Methods:**

This study was informed by the Theoretical Domains Framework and Behaviour Change Techniques Taxonomy to match participant reported determinants of trial retention to theoretically informed behaviour change strategies. The prototype interventions were described and developed in a co-design workshop with trial participants. Acceptability and feasibility (guided by (by the Theoretical Framework of Acceptability) of two prioritised retention interventions was explored during a focus group involving a range of trial stakeholders (e.g. trial participants, trial managers, research nurses, trialists, research ethics committee members). Following focus group discussions stakeholders completed an intervention acceptability questionnaire.

**Results:**

Eight trial participants contributed to the co-design of the retention interventions. Four behaviour change interventions were designed: (1) incentives and rewards for follow-up clinic attendance, (2) goal setting for improving questionnaire return, (3) participant self-monitoring to improve questionnaire return and/or clinic attendance, and (4) motivational information to improve questionnaire return and clinic attendance. Eighteen trial stakeholders discussed the two prioritised interventions. The motivational information intervention was deemed acceptable and considered straightforward to implement whilst the goal setting intervention was viewed as less clear and less acceptable.

**Conclusions:**

This is the first study to develop interventions to improve trial retention that are based on the accounts of trial participants and also conceptualised and developed as behaviour change interventions (to encourage attendance at trial research visit or return a trial questionnaire). Further testing of these interventions is required to assess effectiveness.

**Supplementary Information:**

The online version contains supplementary material available at 10.1186/s13063-022-06218-8.

## Background

A failure to recruit or retain the required numbers of participants in a trial can render the findings unreliable or unusable. Unsurprisingly, recruitment and retention in clinical trials have been identified as the top priorities for methodological research by UK Clinical Trials Unit Directors [1]. Yet, there is an imbalance in the focus of existing research on how both, with recruitment research having approximately four times as many publications as those on retention (data taken from a search of the a recent search of the Online Resource for Research in Clinical triALs (ORRCA project) [2]. Whilst there is clearly a need for methodological research on trial recruitment, the requirement to ensure that once recruited we do not lose people from any stage of our trials also requires attention. For example, an investigation of UK NIHR Health Technology Assessment funded trials found that approximately 50% of trials lost over 11% of follow-up data and some suffered loss to follow-up of up to 77% [3]. It is essential that we ensure the efforts put into recruiting participants are not diluted through a trials failure to retain them.

Many aspects of trial process can be considered as a behaviour, that is, people performing an action (or not), and this is particularly true for retention. Whilst framed as ‘retention’ from a trialist perspective, a more participant focused behavioural description would be ‘sustained participation’. For example, participants are asked to complete and return questionnaires and may be asked to attend research visits in hospital or other research sites, both of which are behaviours that require sustained participation. Many factors influence whether individuals successfully perform these desired behaviours. Importantly, some of these factors and behaviours are also amenable to change. The application of behaviour change theories and frameworks can provide a structure for systematically identifying determinants that are amenable to change and developing evidence-based, theory-guided interventions to target the identified modifiable determinants. Therefore, conceptualising retention as a specific behaviour(s) allows for existing theories and frameworks to be applied to help investigate the problem. Understanding what motivates participants decisions (and ultimately behaviours) to complete a trial has been identified as the top priority for methodological research on trial retention [4].

The application of behavioural science to health seeks to use the theories, methods and existing evidence from a range of disciplines to design more effective heath care interventions. To date, the research relating to recruitment and retention in clinical trials has tended to focus on a handful of theories or frameworks [5]. One of the frameworks applied more recently is the Theoretical Domains Framework (TDF), which summarises 33 behaviour change theories (and therefore offers a range of modifiable factors linked with behaviour and behaviour change) into 14 theoretical domains [6]. Once priority theoretical domains have been identified using the TDF (which could be done through a range of methods), they can be targeted during intervention development by incorporation of behaviour change techniques (BCTs) [6]. BCTs are defined as the smallest ‘active ingredient’ of an intervention and can be used alone or in combination with other BCTs to target key behaviours [7]. A taxonomy of these 93 BCTs has been generated (through international consensus) that can be applied to systematically describe, review and replicate core content of new and existing behaviour change interventions [7]. Hypothesised links between BCTs and mechanisms of action (the process through which participants change behaviour) can then be tested through evaluation of said interventions [8].

The positive effects of a behavioural approach to intervention development has been well evidenced in other patient behaviours (e.g. smoking cessation) and more recently has shown promise for trial retention [9, 10]. For example, behaviour change techniques to encourage participants to return questionnaires have been embedded within cover letters accompanying follow-up questionnaires and shown to increase response rates [10]. However, the adoption of this behavioural approach is the exception rather than the norm. In the most recent update to the Cochrane review on interventions to improve retention to trials, nearly all of the interventions included fail to report the use of theory in their development, with no pre-specified logic model or mechanism of action considered for any potential effect on (behaviour related to) retention [11]. Questions about the adequacy of the design and development of existing retention interventions have been raised by the lack of high quality evidence identified in the Cochrane review [11].

In addition to a lack of theorical input into the development of these existing retention interventions, there has also been a lack of patient and/or participant input, or at least, a lack of reporting that this took place [11]. Furthermore, existing interventions do not address the challenges that participants report as affecting retention in trials. An evidence synthesis of qualitative studies that explored reported reasons relating to trial retention highlighted the ‘fit’ of the trial with participants’ personal beliefs, preferences, capabilities and life circumstances as being key [12]. There are no interventions within the existing Cochrane review that would address these reported priorities. In addition, whilst centring the development of these interventions in accounts of trial participants, it is also important to explore intervention acceptability amongst a range of trial stakeholders who would be tasked with their implementation.

In summary, current interventions do not explicitly address the challenges with retention that participants report nor has behavioural theory been widely applied during the development of such retention interventions. The aim of this study was to tackle these problems by developing and assessing the acceptability amongst stakeholders of participant-centred, theory-informed interventions to improve trial retention.

## Methods

**Fig. 1:**
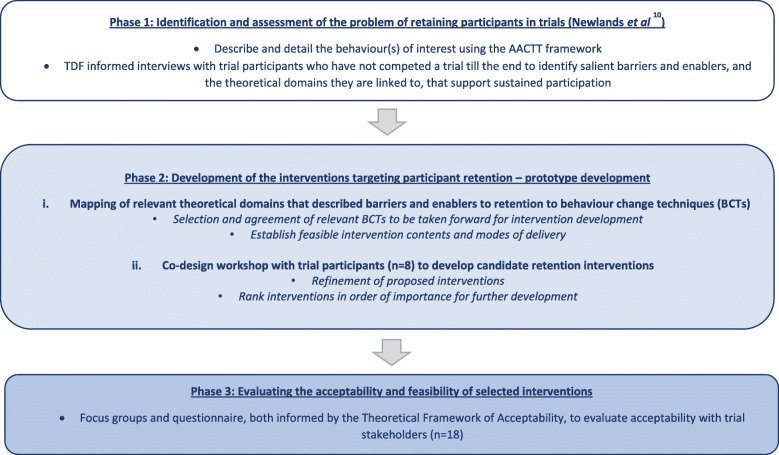
Outline of the process for development the theory-informed, participant-centred, retention interventions

The STEER project (Systematic Techniques to Enhance rEtention in Randomised controlled trials) included a multi-phase mixed methods approach to identifying behavioural barriers and enablers to trial retention and then developing targeted evidence-based solutions to overcome them [13]. Figure 1 outlines the process used to develop and perform preliminary evaluation of the interventions.

### Phase 1: Identification and assessment of the problem

To develop the interventions we first specified the target behaviours using the Action, Actor, Context, Target, Time (AACTT) framework [14] and focused on participant retention in trials requiring questionnaire return and/or attendance at follow-up clinics (see Supplementary Information Table 1). ACCTT provides a framework to describe and detail target behaviours and serves, amongst other functions, to enhance the specificity of assessment of theoretical constructs and behaviour, which can inform development of research tools (e.g. topic guides) and process/outcome measures. Next, we applied the TDF to identify the barriers and enablers of the target behaviours and to identify and assess the problem to directly inform and guide the choice of intervention components. The TDF domains identified as being key barriers or enablers of the target behaviour were identified in a qualitative study reported elsewhere [15]. This identification of priority domains to target with behaviour change interventions identified seven domains that were relevant for both attending clinic appointments for research and returning postal questionnaires and these were taken forward to develop interventions that could target both behaviours. Of these areas of overlap, evidence already existed to support the use of incentives and rewards (reinforcement) for questionnaire return, and so, there was a logical progression to extend this to the alternate behaviour of clinic attendance. Finally, two domains were identified as relevant for questionnaire return only which led to the development of one intervention (focussed on the goals domain) targeting questionnaire return only.

This paper reports the development of the interventions from the stage of identifying intervention components and assessing their acceptability and feasibility.

### Phase 2: Development of the interventions targeting participant retention

i. Mapping of relevant theoretical domains that described barriers and enablers to retention to behaviour change techniques (BCTs)

The first stage in the development of the retention interventions was to identify intervention components to target the relevant TDF domains encompassing relevant barriers or enablers to retention that had been identified in the earlier behavioural investigation [15]. Intervention components were determined using a standardised process that involved mapping the relevant theoretical domains to BCTs using the Theory and Techniques Tool [16, 17]. BCTs are defined as the smallest active ingredient of an intervention such as goal setting or self- monitoring of behaviour, and they can be used alone or in combination with other BCTs [7]. The potential BCTs linked to selected TDF domains were first identified (by RN) and discussed with three other researchers (ED, JP, KG) to reach an agreement about which BCTs were to be taken forward to be developed into an intervention. BCTs which were agreed as not relevant to the target behaviour were excluded. Next, the content of the intervention (based on selected BCTs that will be delivered to overcome the modifiable barriers and/or enhance the enablers) and possible modes of delivery (how each chosen technique would be delivered) were established through discussion (RN, ED, KG). The final selection of intervention content and mode of delivery was considered based on what was practically relevant, likely to be feasible, and could be implemented as a cohesive intervention.

ii. Co-design workshop for developing candidate retention interventions

The aim of this stage was to involve trial participants in further developing the proposed interventions, with the specific goal of ensuring chosen intervention packages were fit for purpose from the perspective of those who have the lived experience of taking part in a trial. Participants from the phase 1 interview stage of the project [15] who had provided consent to be contacted for future studies were invited to take part in a co-design workshop. After initial contact, a researcher (RN) contacted the interested participants to discuss the study further. If willing to participate, each participant was mailed a study pack (including study summary, agenda, visual summary of potential interventions, all produced with the help of the Public Partners) in advance of the workshop. Written consent was sought and documented, by RN, before the start of the meeting.

**Fig. 2:**
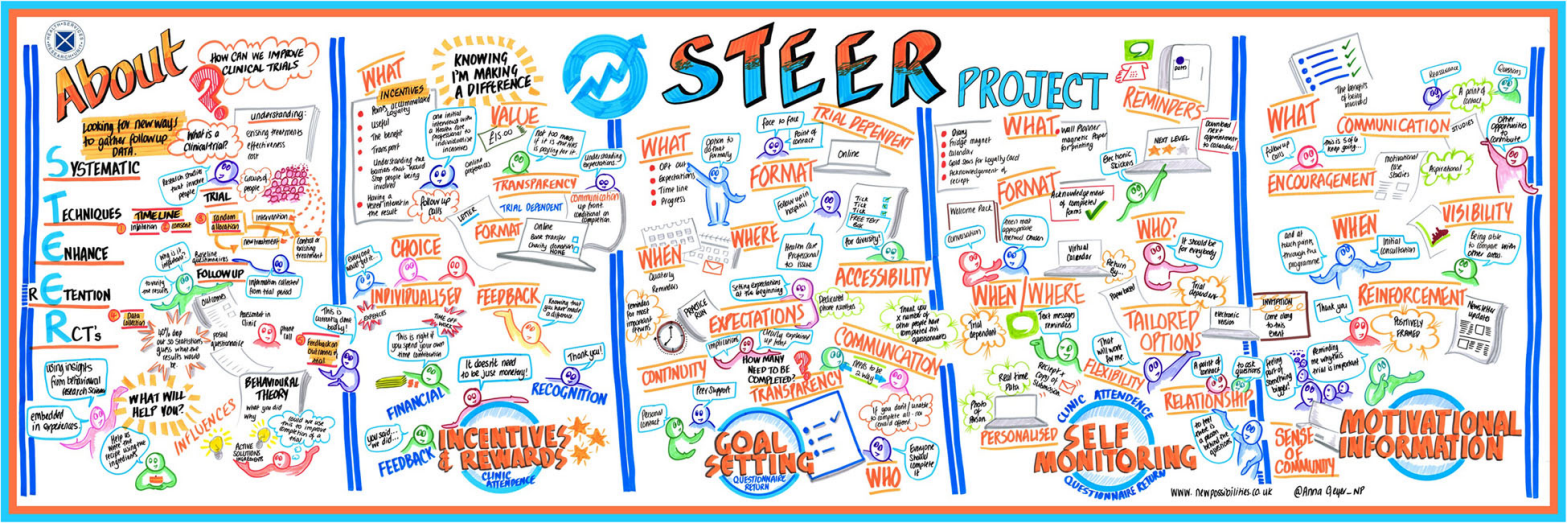
Visual presentation of the co-design workshop discussion

The workshop took place on 4 June 2019 at the Health Research Authority (HRA) Business Delivery Unit in London. The workshop started with a plenary session that covered a summary of the STEER project to date, aims of the workshop, and presentation of the potential interventions (led by KG). Participants were then divided into two smaller groups and asked to discuss each of the potential retention interventions. Discussion was encouraged to be broad and open initially, and then groups were asked to focus on: what should be included in each intervention, what format should each approach take, when should it be delivered, where should this take place, how often should it be delivered, who should deliver it, and who should receive it. The small groups were then brought back together to share the main points highlighted and encourage further discussion. Four researchers (KG & ST (trials methodologists) and ED &RN (implementation scientists) and a Public Partner (JE) facilitated the group discussions. All discussions were recorded using a digital audio recorder and transcribed verbatim. Discussions were also documented in real-time through graphic illustration by a professional graphic illustrator (Fig. 2).

Directed content analysis, focussing on participant-reported concerns and/or advantages related to each intervention, was performed (RN) [18]. The summarised findings were checked by another member of the research team (KG) by comparing findings to original transcripts and notes taken during the meeting (by ED and ST). A summary of the discussion of each intervention was sent to participants along with a copy of the graphic illustration for any further feedback. Participants were also sent a short questionnaire asking them to rank the interventions in order of importance for future development and testing (which was informed by conversations in the co-production workshop where participants considered importance against how likely the intervention may impact their behaviour, i.e. perceived effectiveness).

### Phase 3: Evaluating acceptability and feasibility of selected interventions

The next phase of the intervention development process involved assessing the acceptability and feasibility of the two priority interventions (selected by co-design workshop participants) using in person focus groups. Participants for these were sought from a range of stakeholder groups: potential trial participants, trial staff, and Research Ethics Committee (REC) members. Potential trial participants were invited from the list of participants who had taken part (or indicated interest) in earlier phases of the study. Trial staff were invited through known contacts of the research team. REC members were invited through known contacts of the research team and by direct invitation to REC committees based near the meeting location.

The in-person meeting took place in Birmingham, UK, on the 2 September 2019. The meeting started with a presentation to provide an overview of the project and the proposed interventions (focussing on content, mode of delivery and contextual applicability). Participants were divided into two groups (balanced by stakeholder group) and asked to discuss each intervention. Discussion was guided by a semi-structured topic guide and each group facilitated by members of the research team (RN, KG, ED, ST). The topic guide was developed in collaboration with the Public Partners and was informed by the Theoretical Framework of Acceptability (TFA [19], see Supplementary Information Table 2). The TFA was developed specifically to assess the acceptability of health care interventions and is composed of seven interrelated constructs: affective attitude, burden, perceived effectiveness, ethicality, intervention coherence, opportunity costs, and self-efficacy [16]. The use of this framework allows for more robust and reproducible assessments of intervention acceptability. Following small group discussion, each group fed back to the whole group for further discussion. All discussions were audio recorded and transcribed verbatim. At the end of the meeting participants were requested to complete and return an anonymous TFA-informed questionnaire (with questions framed around the seven constructs) to record the extent of their agreement with the expressed views of the majority or to state anything that they were not able to voice in the group. Two TFA questionnaires were developed which were identical in their construct content but where the question framing was tailored for stakeholder groups, i.e. one was targeted to those who would receive the intervention (e.g. trial participants and REC members) and others who would deliver the intervention (e.g. trial teams). Descriptive statistics (i.e. median) were used to analyse questionnaire responses. The qualitative data from the focus groups were analysed using a deductive directed content analysis approach [18] with the qualitative data providing further detail to survey responses.

### Patient and public involvement

Two Public Partners (J.E. and M.O.) were part of the study team and involved at several stages including the development of study protocol, the design of the study materials (e.g. invitation letter, information leaflet), ethics application, and discussion at the steering committee’s meetings. They also had considerable input into the phrasing of the topic guide and survey questions as well as planning and conducting meetings with research participants. Our public partners have extensive experience of working across a range of health research projects, funding panels, and policy roles (e.g. patient and public involvement lead for the Health Research Authority) and so have an in depth understanding of randomised trials.

## Results

### Phase 2: Development of the interventions targeting participant retention—prototype development

i. Mapping of relevant theoretical domains that described barriers and enablers to retention to behaviour change techniques (BCTs)

Table 1 provides details of the BCT mapping and intervention development stage. The columns indicate how the relevant identified theoretical domains were linked to selected BCTs to target barriers and enablers, and then intervention contents and modes of delivery proposed. A total of four candidate interventions were generated addressing one or both target retention behaviours (questionnaire return, clinic attendance).

ii. Co-design workshop for developing candidate retention interventions

**Table 1:** BCT mapping to develop interventions to improve participant retention in trials

Intervention 1: Incentives or rewards to improve trial follow-up clinic attendance *Target behaviour = follow-up clinic attendance*				
TDF domains (frequency*)	*Linked BCTs (to be taken forward)*	*Behaviour change objectives*	*Example quotes to illustrate the beliefs relevant for the TDF domain identified*	*Possible intervention content*
*Reinforcement (7/7), Beliefs about consequences (7/7) and* *Social influences (6/7)*	**10.1.** Material incentive (behaviour) **10.2** Material reward (behaviour) **10.3** Non-specific reward **10.6** Non-specific incentive**10.8.** Incentive (outcome) **10.10** Reward (outcome) **10.4** Social reward	Inform the participants that a reward (money/vouchers/other valued objects) will be delivered if and only if there has been effort and/or progress in performing the behaviour. Arrange for the delivery of a reward (verbal/non-verbal/money/vouchers/other valued objects) if and only if there has been effort and/or progress in performing the behaviour.	*Yes, incentives are always good aren’t they? Like shopping vouchers or cash, or.* *That was very much appreciated. … it’s like a little voucher to say thank you for participating, and that was good. I think the other thing that would be encouraging as I mentioned before, is when the survey is completed just to have a little note with a few bullet points on about what the findings from the survey were …* *I… when I returned the questionnaire it would’ve been nice to receive a small note saying, “Thank you Mr X, we’ve received the questionnaire and it’s going to be included as part of the study”, or just a little recognition that the document had been received and it was now going to be processed as part of the study.*	Send an email/letter which thanks them for their time to take part in the trial and/or contains a voucher code and instructions on how to claim it.
**Intervention 2: Goal setting for improving questionnaire return** *Target behaviour = questionnaire return*				
*TDF domain* (frequency*)	*Linked BCTs (to be taken forward)*	*Behaviour change objectives*	*Example quotes to illustrate the beliefs relevant for the TDF domain identified*	*Possible intervention contents*
Goals (13/16)	**1.1** Goal Setting (behaviour)	Set or agree on a goal defined in terms of behaviour to be achieved	*As for the survey they sent me questionnaires through the post, I haven’t done that because I am on family commitments. … It’s probably about middle priority.* *it’s a job I have to do. In terms of where it fits with what’s going on in my life with the kids, with work, it’s probably not as high up as that but it’s definitely a job I know I have to complete and send back.*	Set goals/targets with participants (during consenting process) that all (e.g. six out of six) questionnaires to be returned to complete taking part in a trial.
**Intervention 3: Self-monitoring to improve questionnaire return and clinic attendance** *Target behaviour = follow-up clinic attendance and questionnaire return*				
TDF domain (frequency*)	*Linked BCTs (to be taken forward)*	*Behaviour change objectives*	*Example quotes to illustrate the beliefs relevant for the TDF domain identified*	*Possible intervention contents*
Behaviour regulation (clinic attendance 7/7 and questionnaire return 14/16)	**2.3.** Self-monitoring of behaviour	Establish a method for the participants to monitor and record their behaviours (i.e. attending clinic appointments/returning questionnaires) as part of a behaviour change strategy to reinforce retention behaviour.	*I always had it out in a visible place, so it was always on my coffee table. A visual reminder.* *I tend to use that (iPhone and a calendar) quite a lot as a reminder* *I always make sure that I’ve got it (date and time of a clinic appointment) in my diary, and I allocate the time.*	Give participants a chart/worksheet/trial calendar of all activities (e.g. how many questionnaires to complete and return and by when). Additionally, a sticker (stating the number of activities out of total activities to be completed) could be sent out with the invitation letter to put on the self-monitoring chart once the task is completed.
**Intervention 4: Motivational information to improve questionnaire return and clinic attendance** *Target behaviour = follow-up clinic attendance and questionnaire return*				
*TDF domain (frequency)*	*Linked BCTs (to be taken forward)*	*Behaviour change objectives*	*Example quotes to illustrate the beliefs relevant for the TDF domain identified*	*Possible intervention contents*
Beliefs about consequences, (clinic attendance 7/7 and questionnaire return 15/16)	**5.1**. Information about health consequences **5.2**. Salience of consequences **5.3.** Information about social and environment consequences	Provide information (e.g. written, verbal, visual) about health/social/environmental consequences of performing the behaviour. Use methods specifically designed to emphasise the consequences with the aim of making them more memorable (goes beyond informing about consequences)	*I suppose it is a benefit if I’m able to help in the study, if my contribution helps in any way then that’s a benefit to me as well, I suppose.* *Nothing, I suppose. I didn’t think anything would happen [if they didn’t return the questionnaire].* *If they don’t do it, they might upset the trials, and just make a loss of money doing the trial.*	Persuade participants to complete behaviour by providing information about consequences of performing a trial related behaviour (clinic attendance/questionnaire return) such as if they do/don’t return the questionnaire what impact it might have on the trial progress/findings. Emphasise this information further with the aim of making them unforgettable to participants.

*Frequency relates to the number of participants reporting barriers or enablers within the domain

A total of 34 potential trial participants were invited to participate in the co-design workshop and eight (23.5%) took part in the in person workshop which lasted 5.5 h. Co-design participants had participated in a range of clinical trials: in oncology, oral health, mental health, and women’s health, which were evaluating a range of clinical interventions (Table 2). The target retention behaviours within these trials included return of postal or online questionnaires and/or clinic visits. Overall, participants reported that all four of the proposed interventions were important and could be delivered to all trial participants (i.e. both those who have and have not completed trial retention activities) and adapted based on the design of the original trial e.g. activities to be delivered and received via online. More general findings concerning trial retention were also voiced in the workshop (and echoed the findings of the interviews but were not deemed salient enough to be developed further), with participants reporting the need for key ‘touch points’ between trial office and participants across the timeline of the trial (e.g. communication during recruitment, follow-up, reminders). Building relationships with trial staff was felt to be important to keep them motivated throughout the trial period. Therefore, having details (name, address, email along with a photo) of the ‘point of contact’ (a person who participants have met once in person or online and was the identified contact for any queries issues during the trial) was suggested as essential for maintaining commitment to the trial. Summaries for each intervention as discussed by participants are presented below with quotes from participants presented in italics (see Table 3 and Table 4 for further details).

**Table 2:** Demographic details of phase 2 co-design workshop participants

Trial acronym, ISRCTN, title	Co-design participants	Target behaviour
MASTER: ISRCTN49212975 A UK multicentre RCT evaluating the male synthetic sling versus Artificial urinary Sphincter Trial for men with urodynamic stress incontinence (USI) after prostate surgery	*n* = 2 (males, 56 years and 72 years)	Return of postal questionnaire At 12 and 24 months post-randomisation
DISCO: ISRCTN89237370 A UK online RCT of the effects of digital cognitive behavioural therapy (CBT) for insomnia on cognitive function	*n* = 1 (female, 56 years)	Online return of questionnaires 10 and 24 weeks post-randomisation
INTERVAL: ISRCTN95933794 A UK multicentre RCT investigating the best dental recall interval for optimum, cost-effective maintenance of oral health in dentate adults attending dental primary care	*n* = 4 [2 females (65 years, 71 years), 2 males (58 years, 65 years)]	Return of postal questionnaires and clinic visits - Questionnaire: 3, 6, 12, and 24 months post-randomisation - Clinic visit: 6 months, 24 months, risk-based recall
Various trials in relation to women’s health	*n* = 1 (female, 38 years)	Return of postal questionnaires and clinic visits. Timing unknown

*All participants had been a participant in a trial in the preceding 12 months

**Table 3:** Summary of findings from co-design workshop

Intervention	Content/what	When	Format	Where	How often	Who should provide this?	Who should receive it?
**1: Incentives and Rewards for follow-up clinic attendance**	**a**. Monetary incentive and reward: Financial incentives which participants could choose (e.g. monetary, charitable donation, prefer not to receive). Total incentive cost should not exceed £15, which may be broken down for each visit (i.e. £5 for each visit) that is accrued over time. **b. Non-monetary** (social) reward: Send ‘Thank you’ note after attending each clinic and mention that they are making a difference.	**a.** Monetary incentive and reward: Inform during and after recruitment/ randomisation **b**. Non-monetary (social) reward: At the end of a trial	Trial dependent: written information delivered by post or electronically (i.e. email containing online link for charity/vouchers options)	Participant receives at home	**a.** Monetary incentive and reward: On completion of all visits **b**. Non-monetary (social) reward: After each visit	Trial dependent: trial office/point of contact, health professionals, e.g. clinical specialists	Conditional on behaviour: all participants would have the opportunity to receive the incentive but only those who complete the behaviour would get the reward
**2: Goal setting for improving questionnaire return**	Set goals that all questionnaires need to be returned. Show an example of the questionnaire and provide an opportunity to work through Provide the contact details (and photo) of the point of contact for any queries During follow-up provide number (%) of other people who completed the questionnaire to encourage further	During the beginning of the trial likely during the informed consent process.	Trial dependent: verbal, paper based, electronic.	Trial dependent: at trial site or home.	Dependent on the total duration and how many follow-up points in the trial	Trial dependent: by the point of contact, health professional, recruiter or peer from the trial	All participants
**3: Self-monitoring to improve questionnaire return and clinic attendance**	Two options: 1. Provide a portable sized loyalty card (indicating date when questionnaire returns or clinic visits due). 2. Provide a personalised planner—as above with dates for clinic visit or questionnaire completions. On the other side of the card, mention the purpose of the trial and details/photo of the point of contact Participants will receive a sticker (after completing each activity) to put on the card/planner	Given timing of questionnaires/clinics will likely depend on date of randomisation, this needs to be delivered post-randomisation	Trial dependent: verbal, paper based, electronic	Trial dependent: at trial site and/or home	During each follow-up (e.g. a week before) send a reminder about self-monitoring	Trial office/point of contact	All participants
**4: Motivational information to improve questionnaire return and clinic attendance**	Motivational information framed as positive reinforcement e.g. end purpose of this research, benefits of being involved, and how others are doing in the trial	Initial recruitment consultation, in the patient information leaflet and throughout the trial during any patient contact	Trial dependent: verbal, paper based, electronic	Trial dependent: at trial site and/or home	Dependent on the trial duration e.g. will be linked to key ‘touch points’ between trial office and participants	Trial office/point of contact	All participants
	Case studies: Aspirational messages about how other research has changed clinical practice						
	Online forum/peer support to encourage participants throughout the trial period

**Table 4:** Intervention description summary

***1. Incentives and rewards for follow-up clinic attendance*** *Ideally, participants could choose which financial reward (e.g. monetary, charitable donation/prefer not to receive) they would like to receive within the trial after completing all activities. Receipt of the financial rewards will be dependent on performing the activity, i.e. attending the clinic, and will be received at the final visit based on their overall attendance. Participants should be told during the recruitment discussion about the financial reward and the total amount that they would receive after attending all clinics. Participants should also be told that if they miss some visits, then that amount will be deducted from the total amount. In short, all participants who consented take part would have the opportunity to receive the reward but only those who attend the clinic would get the reward. The total financial reward should be explored with patient partners and should be reflective of the input required.* ***2. Goal setting for improving questionnaire return*** *During the informed consent consultation, provide an example of the questionnaire(s) and offer the potential participant an opportunity to work through. This will provide realistic expectations about what sort of questions will be presented and may help them to keep a record of these from the beginning (e.g. How much bodily pain have you had during the past 4 weeks?). Ask the participant to consider how many of the questionnaires they will commit to returning.* Throughout the trial, participants will be reminded of the ‘goals’ agreed or set to encourage completion of all questionnaires, e.g. When sending the questionnaire, remind participants that during the consent discussion they agreed to complete trial follow-up. ***3. Self-monitoring to improve questionnaire return and/or clinic attendance*** *If the questionnaires are paper based, provide a ‘loyalty’ card (indicating date when questionnaires are to be returned or clinic attendance). The card should be small enough to bring to visits without inconvenience. On the reverse of the card, mention the purpose of the trial and give details/photo of the point of contact. If electronic then provide electronic tracker or outlook planner and reminder. Given that the timing of questionnaires/clinics will likely depend on the date of randomisation, the card/planner will be delivered post-randomisation with key follow-up dates generated accordingly. Participants will receive a sticker (by post or a virtual sticker if by email) to put on the card/planner after the trial office has received each questionnaire, or after a visit has been attended.* ***4. Motivational information to improve questionnaire return and clinic attendance*** Provide motivational information framed as positive reinforcement during the initial recruitment consultation, in the patient information leaflet (and supporting conversation) and throughout the trial during any patient contact or key trial touch points. The contents of the intervention should be tailored based on the trial, its participants and purposes. Remind participants about the focus and purpose of the trial and its possible impacts on future practice/guidelines (i.e., the end goal/bigger picture) and how their contribution is making a difference. You may want to identify the key potential benefits of being a research participant, such as tackling health issues/helping future generation/family members if they need a treatment for the same health condition in the future and that the more people who complete a task, the quicker the trial could help others. Keep thanking people for their contribution, for example, after every returned questionnaire or visit. State how others are doing in that trial for social comparison e.g.’ *About 70% of people have come for this clinic appointment. We need everyone to come along for their visit to make sure that the results of the trial are as scientifically strong as they possibly can be.’* If the participant missed an activity, still encourage them saying they are still in the trial and that their continued contribution is still important and valued.	

### Intervention 1: Incentives and rewards to improve follow-up clinic attendance

For the first intervention, participants were asked to consider monetary (i.e. financial incentive/reward) and non-monetary (i.e. social reward such as a ‘thank you’) incentives and rewards. All participants agreed that there should be acknowledgment of their contributions, through incentives or rewards for completing trial activities because these are motivating, and it would make their contribution to the trial ‘feel valued’. There were mixed opinions regarding what the incentive or reward should be (e.g. shopping vouchers, lottery tickets, thank you note, donate money to either a charity related to the study or one that they wish to nominate). However, they all agreed that the potential for either monetary or non-monetary options should be available, and the choice could be informed by participants’ preferences. With regard to who should provide this intervention, participants commented that this would be dependent on the trial design but was likely to be the trial office, a health professional or a point of contact. They all agreed that participants should be informed at the beginning of a trial about the incentives/rewards available. Participants also discussed what they felt was an acceptable value for the monetary incentives/rewards, which in the context of publicly funded trials should not be ‘too much’ (i.e. cost should not exceed £15). It was proposed that money could be split up into smaller amounts (e.g. £5 for each visit) and could be cumulative, based on retention activity completed. When considering social rewards, such as a thank you note, participants expressed a desire to receive this after each clinic attendance.

### Intervention 2: Goal setting to improve questionnaire return

The second intervention proposed and discussed was the potential for setting goals about returning trial questionnaires. Participants agreed that setting targets at the beginning of their trial participation with trial staff (e.g. a person who seeks consent) is important to create an expectation of activities to be completed during a trial period. Participants reported that a goal-setting intervention for questionnaire return would provide transparency regarding how many questionnaires are to be completed, highlight that some may be repetitive, make clear when they would receive them, and, finally, give an indication of how long it might take to complete one. They wanted to receive reminders (e.g. during follow-up points) of the set goals and where possible, the number (as a percentage) of other participants who have completed the questionnaire at that time point, to encourage completion of all questionnaires. Some agreed that ‘a contract should be signed’ between the trial office and participants, but others noted the need for flexibility during participation if personal or life or health circumstances changed.

### Intervention 3: Participant elf-monitoring to improve questionnaire return and/or clinic attendance

The third intervention proposed was a self-monitoring intervention in the form of a card or personalised planner that would indicate when the questionnaires were due. Once a retention behaviour had been completed, participants would receive a sticker/stamp to indicate the behaviour had been completed. A range of items to support self-monitoring (trial-specific calendar, electronic invitation to connect with online calendar, fridge magnets, diary, loyalty card) were suggested by participants that could highlight the dates regarding when clinic visits or questionnaire returns are due. However, participants agreed that a portable sized loyalty card or a planner should be provided after randomisation so that these items could be personalised for specific follow-up time points. It was suggested that on each side of the card or planner some information could be added such as due dates of activities, the purpose of the trial and details of the point of contact. They would have liked to receive reminders regarding self-monitoring from the trial office at each follow-up time. For further reinforcement, some participants in our sample suggested that a sticker (in the format of a gold star, electronic trophies, or something similar) could be sent out by the trial office as an acknowledgement after completing each activity, to insert on the card/planner.

### Intervention 4: Motivational information to improve questionnaire return and clinic attendance

The fourth intervention proposed motivational information to target retention behaviour. Participants believed that providing aspirational messages about how other research has changed clinical practice, and the purpose and benefits of taking part in the trial, would motivate their retention behaviour. These interventions would be delivered during key ‘touch points’ between the trial office and participants (e.g. communication during recruitment, follow-up, reminders). Various modes of delivery were discussed amongst the groups including incorporating this information into welcome packs, invitation letters, newsletters, posters, text messages, email, telephone communication. Whilst more social reward than motivational information, it was also suggested by participants that arranging events (e.g. inviting participants to trial conferences, meetings, Christmas meals) ‘to feel part of the trial family’ would be beneficial to keep participants motivated throughout the trial period. Furthermore, arranging events (online or in person e.g. coffee mornings) with other trial participants was suggested as desirable for peer support during the trial period.

Participants reported that the incentives/rewards intervention and the self-monitoring of behaviour were straightforward to deliver and as such were not prioritised for further development. The goal setting and motivational information interventions were selected by the participants for further exploration during the acceptability and feasibility focus group.

### Phase 3: Evaluating the acceptability and feasibility of selected interventions

This phase of the intervention development process included focus group meetings where participants were asked to contribute to discussions and then to independently complete an anonymous questionnaire to score the proposed interventions on completion of the discussion. A total of 18 participants (33.3% out of 54 people invited) took part in the focus group meetings and completed the associated questionnaire (Table 5). Two focus groups were run concurrently and lasted 4 h in total. The two groups each included eight participants from mixed stakeholder groups. Participants covered a range of trial roles including trial participants, trial managers, database managers, Clinical Trials Unit Directors, research nurses, and ethics committee members. All trial staff reported in the questionnaire that they ‘strongly agreed’ that improving retention of participants in clinical trials is something they care about and see as part of their role. Views and perspectives of trial staff (who would be delivering retention interventions) and of trial participants and ethics committee members (who would be receiving retention interventions) are presented below, for each intervention (see Table 6).

**Table 5:** Demographic of the phase 3 focus group participants

		Number of participants (***N*** = 18)
*Role*	Trial participant	4
	PPI member	1
	Clinical trial unit director	2
	Trial manager	2
	Database manager	1
	Research nurse (various fields)	3
	Research midwife	1
	Research ethics committee members	4
*Gender*		10 female/8 male

**Table 6:** Results of phase 3 questionnaire assessing acceptability of interventions

Questions	Intervention deliverers: trial staff (***n*** = 9) *group median*	Intervention receivers/regulators: trial participants and REC members (***n*** = 9) *group median*
Improving retention of participants in clinical trials is something I care about	Strongly agree	
I have a role to play in helping to improving retention of participants in clinical trials	Strongly agree	
*Motivational information*		
How likely is the intervention will improve retention? *(TFA construct: perceived effectiveness)*	Somewhat likely	Extremely likely
How do you feel about delivering/receiving the intervention? *(TFA construct: affective attitude)*	Strongly likely	Likely
How much effort would be required to deliver/engage with the intervention? *(TFA construct: burden)*	Some effort	A little effort
Do you think delivering/engaging with this intervention would interfere with other things you need to do? *(TFA construct: opportunity cost)*	Disagree	Slightly
How confident are you that you will be able to deliver/engage with this intervention? *(TFA construct: self-efficacy)*	Very	Confident
Is it clear to you how the intervention would be delivered and received/how it might encourage participants to improve questionnaire return or clinic attendance? *(TFA construct: intervention coherence)*	Clear to very clear	Somewhat
How likely is it that you would use this intervention in practice? *(TFA construct: intervention coherence)*	Very likely	–
Do you think it will be ethical to use this intervention? *(TFA construct: ethicality)*	–	Strongly Agree
*Goal setting*		
How likely is the intervention will improve retention? *(TFA construct: perceived effectiveness)*	Somewhat likely	Likely
How do you feel about delivering/receiving the intervention? *(TFA construct: affective attitude)*	Likely	Likely
How much effort would be required to deliver/engage with the intervention? *(TFA construct: burden)*	A lot of effort	Huge effort
Do you think delivering this intervention would interfere with other things you need to do? *(TFA construct: opportunity cost)*	No opinion	Moderately
How confident are you that you will be able to deliver/engage with this intervention? *(TFA construct: self-efficacy)*	Somewhat	Confident
Is it clear to you how the intervention would be delivered and received and how it would work to improve questionnaire return or clinic attendance? *(TFA construct: intervention coherence)*	Somewhat unclear/clear	Somewhat
How likely is it that you would use this intervention in practice? *(TFA construct: intervention coherence)*	Likely	–
Do you think it will be ethical to use this intervention? *(TFA construct: ethicality)*	–	Agree

– = question not asked to this group

For both goal-setting and motivational information there were discussions about the timing of delivery. Participants commented that a lot of the activity would have to be ‘front-loaded at recruitment’ and then emphasised throughout, recognising this requires commitment and resource from the trial teams (TFA construct: burden). They recognised that currently much of the training of staff at trial sites focuses on recruitment, with not much attention given to follow-up or what to do if someone changes their mind during a trial—often due to the incentivisation for recruitment over retention. It was acknowledged that ‘front-loading’ discussions about retention in time-critical settings (e.g. emergency departments or labour wards) is likely to be challenging (TFA construct: opportunity cost). Furthermore, trials involving patients with poor prognosis or high mortality risk (e.g. some cardiovascular conditions and cancers) might require a different approach (TFA construct: ethicality).

### Motivational information to improve questionnaire return and clinic attendance

Overall, both groups scored the questionnaire item about motivational information intervention improving trial retention as ‘somewhat or extremely likely’ (TFA construct: perceived effectiveness). There were various mentions in the focus groups of the perception that this type of motivational information is being ‘used routinely anyway’. A key recommendation was that the motivational information should be specific to the trial and be relevant to the individual without becoming too repetitive or insincere (e.g. ‘thank you’ at multiple time points). If the information about retention was given (verbally or in writing) during recruitment, it was felt by some that it would need to be given separately from information on the participant’s right to withdraw (TFA construct: intervention coherence). Effective communication was mentioned by many focus group participants as being key for this intervention.

With regard to the effort required to interact with this intervention, questionnaire responses highlighted that trial staff reported ‘some effort’ would be required whilst trial participants and REC members reported ‘little effort’ (TFA construct: burden). When asked to score in the questionnaire how confident they were that they could deliver or engage with the intervention focus groups participants were positive (TFA construct: self-efficacy), which matched how they felt about the intervention overall (TFA construct: affective attitude). Some suggested that digital techniques, e.g. apps could enhance engagement with motivational interventions, but there was an acknowledgement that this might exclude some patients, particularly those who are already at risk of not being retained in the trial (TFA construct: ethicality).

Trial staff said it was very likely that they would use this intervention in practice, with participants in the focus groups reporting that they felt it would be easy and straightforward to implement (TFA construct: self-efficacy). Many reported that this type of activity already. Finally, the trial participant questionnaire responders (which included ethics committee members) strongly agreed that this intervention was ethical. However, one of the focus group participants (an ethics committee member) expressed concerns around ensuring the language was not be seen as being coercive, especially if a participant had made a request to withdraw (TFA construct: ethicality).

### Goal-setting to improve questionnaire return

When considering the goal-setting intervention, whilst both groups thought it was likely or somewhat likely to improve retention (TFA construct: perceived effectiveness) and generally felt positive about delivering or receiving this intervention (TFA construct: affective attitude), there were some concerns, particularly around the importance of context*.* For example, providing verbal or written information at recruitment might work for some trials (e.g. of elective surgical procedures) but not for other trials (e.g. of women in labour), for whom a protracted conversation about goal setting would not be feasible. Participants scored in the questionnaire that they were ‘unclear’ about how this intervention would work (TFA construct: intervention coherence) and were ‘less confident’ about delivering or engaging with this intervention (TFA construct: self-efficacy). This is likely linked to questionnaire respondents reporting ‘considerable effort’ being required to deliver or engage with the intervention (TFA construct: burden).

Within the focus groups, participants reported that the terminology of ‘goal setting’ may not be helpful and further articulation was required. It was also felt that if trying to set goals and develop action plans with people, it was important to understand their motivations for trial participation. Again, slightly less enthusiasm was reported by trial staff in the questionnaire responses with regard to them being likely to use this intervention (TFA construct: self-efficacy). Both trial participants and REC members agreed in the questionnaire that this intervention would be perceived as ethical (TFA construct: ethicality). However, there was some scepticism raised in the focus group discussions as to whether or not goal setting was ‘the right thing to do’ from an ethical perspective given it may undermine a trial participants voluntariness.

## Discussion

In this paper, we have presented a detailed report of the development and evaluation (of the acceptability and feasibility) of participant-centred behaviour change interventions to improve retention of participants in trials. This research developed four co-designed behaviour change interventions which could be evaluated in future trials to assess their impact on trial retention.

Historically, many of the interventions targeting trial retention have been developed without reference to an in-depth assessment of the participant-reported challenges to retention. Our retention interventions are amongst the first to be developed that are explicitly embedded in trial participants’ accounts of the barriers and enablers to trial retention. Further, our study involved trial participants through all stages of developing the interventions including the co-design of prototypes (which allowed additional inclusion of linked relevant BCTs) and input into the accessibility and feasibility. There is preliminary evidence that (in a treatment setting) co-designed interventions may be more effective that their usual care comparator providing further support for similar approaches [20]. Therefore, the evaluation of these co-designed interventions to improve retention is a key next step to determine their effectiveness both compared to standard follow-up but also compared to interventions that have not been co-designed.

In addition to the participant-centred nature of the interventions, most existing retention interventions have not been developed within a theoretical framework and are likely to be based on experience and pragmatism. The step-wise approach outlined in this paper begins with behavioural ‘diagnosis’ of the problem by assessing who needs to do what differently and follows through to providing behavioural ‘solutions’ and exploring their acceptability and feasibility with stakeholders. This theory-informed approach, grounded in empirical research, provides a systematic, transparent, process for identifying and determining intervention components and aligns closely with the MRC’s guidance on the development and evaluation of complex interventions [21]. In addition, it should allow more substantive conclusions to be made about hypothesised mechanisms of change that are related to the proposed interventions.

There are emerging examples in the literature of (components of) similar behavioural approaches being used to target trial recruitment to diagnose the problem but also some that extend to the development of theory-informed interventions, in this case targeting urologists to recruit from underserved populations in oncology [22–25]. Encouraging other trial teams to conceptualise trial problems as behaviours and develop behaviour change interventions targeting trial retention will allow the generation of a cumulative evidence base. However, appropriate evaluation of these approaches in a trial context is required to understand whether the evidence from changing health behaviours also transfers to changing trial behaviours. In addition, there is work ongoing that aims to consider the ethical implications of developing and applying behaviour change interventions in the context of clinical trial participation [Professor Charles Weijer personal communication].

### Strengths/limitations

Although we involved stakeholders across the intervention development stages, they were few in number and as such our findings are based on the opinions and perspectives of a relatively small sample and who may already be more engaged in research than other research participants. However, numbers were in line with guidance on sample size requirements for focus groups [26]. In addition, whilst we sampled for diversity in type of trial, we did not sample for diversity with regard to ensuring representation of under-served populations (i.e. ethnic minorities and socioeconomically disadvantaged) [27]. Therefore, we cannot conclude that these interventions would be acceptable to everyone we hope to retain in our trials. We chose to focus on trial retention in phase III pragmatic effectiveness trials and therefore our findings may not be applicable to earlier phase trials assessing efficacy of interventions, which may face different challenges in retaining participants.

A key strength of this study was its involvement of a range of stakeholders (including trial managers, recruiters, and ethics committee members) but most notably, trial participants, at every stage of intervention design and development. In addition, this multi-stakeholder approach also enabled perspectives from multiple trials covering a range of clinical areas and associated clinical interventions. These multiple perspectives allow considerations of context to directly feed into and inform the development of the interventions our research proposes. A further strength of the study was that all aspects were informed by relevant theory. In addition to the TDF-informed interviews to identify the key behavioural targets, the assessment of acceptability of the intervention was also informed by the Theoretical Framework of Acceptability.

Finally, over and above the specific retention interventions we present, the process we describe provides a framework for others to use to develop their own participant-centred, theory-based, interventions for retention and other process challenges that their trials may face. We hope the use of such a framework will help to avoid trial process interventions developed on hunches alone and which have no clear rationale for how they may achieve the desired effect.

## Conclusion

This is the first study to apply a theory lens to the development of interventions to improve trial retention, based on the accounts of trial participants, and other stakeholders. Evaluation and implementation of the interventions in future trials may enable participants to complete a trial until all data collection has finished. Future evaluations of these interventions using embedded Study Within A Trials (SWATs) should also consider the impact on trial staff and other resource implications. In addition to the developed interventions, this study provides a framework for trial teams to develop participant centred, theory based, interventions that could be applied to other problems of trial process and conduct that are behaviourally determined.

## Supplementary Information

**Additional file 1: Table 1.** AACTT specification of participant behaviour. **Table 2.** Topic guide for acceptability/feasibility focus groups in Phase 3. **Table 3.** Quotes from participants in the intervention co-production workshop. **Table 4.** Quotes from trial stakeholders in focus groups exploring intervention acceptability.

## Data Availability

We did not seek consent from participants to share data outside of the research team or for purposes unrelated to this study. As such, only the participant data presented in the published manuscript is available.
