# Supplementary material for: The development of theory-informed participant-centred interventions to maximise participant retention in randomised controlled trials
Source: Trials. 2022 Apr 8;23:268. doi: 10.1186/s13063-022-06218-8 (PMC8994320; doi:10.1186/s13063-022-06218-8)
Supplement: Supplementary file 1 — Additional file 1: Table 1. AACTT specification of participant behaviour. Table 2. Topic guide for acceptability/feasibility focus groups in Phase 3. Table 3. Quotes from participants in the intervention co-production workshop. Table 4. Quotes from trial stakeholders in focus groups exploring intervention acceptability. [file 13063_2022_6218_MOESM1_ESM.docx]

**Supplementary Information**

**Table 1. AACTT specification of participant behaviour.**

| **AACTT** | **Trial participant behaviour** |
| --- | --- |
| Action | Return of questionnaire or attendance at clinic |
| *Actor* | Trial study staff or trial site staff or clinical staff |
| *Context* | At home or in clinic |
| *Target* | All trial participants |
| *Time* | Dependent on trial follow-up time points |

**Table 2: Topic guide for acceptability/feasibility focus groups in Phase 3.**

| **Constructs (TFA)** | **Applicable to: Research Ethics Committee (REC) and Patients (receiving the intervention), Trial staff and Research nurses (delivering the intervention)** | **Prompts (specific to group, if any)** |
| --- | --- | --- |
| Affective attitude (anticipated/experienced): how an individual feels about the intervention prior to taking part | 1. How would you feel about this intervention to encourage participants returning questionnaires/attending clinic appointments? 2. Why do you feel that way? | What are your views about this intervention from the perspectives of  -delivering it/enabling its delivery? (trial staff/RNs)  -receiving the intervention? (participants/REC) |
| Ethicality: the extent to which the intervention has good fit with an individual’s value system | 1. Are there any ethical considerations?   What are they?  [e.g. does it feel coercive? Or  Undue inducements: “…are excessively attractive offers that lead people to do something to which they would normally have real objections based on risk or other fundamental values]  b. What do you think the benefits and disadvantages of this intervention would be? | - related to delivery of this intervention? (trial staff/RNs)  - related to patients receiving it? (participants/REC)  From the perspectives of  -delivering it/enabling its delivery? (trial staff/RNs)  -receiving the intervention? (participants/REC) |
| Intervention coherence: the extent to which the participant understands the intervention and how it works | 1. Could you describe what the intervention is and how it might work? | To what extent do you understand the mechanism/technique of this intervention that should have an impact on retention? |
| Burden: (anticipated/experienced): the amount of effort that is required to participate in the intervention | 1. In your opinion, do you think receiving this intervention would require much work? (participants/REC)   In your opinion, do you think delivering the intervention would require much work? (trial staff/RNs)   1. Any suggestions regarding how this burden/workload could be reduced? | (Time to read information/send extra materials on top of what you normally do)  What would be the acceptability if (intervention- Goal setting)   1. Who: the point of contact (trial team), Health Professional, recruiter or peer from the trial deliver this intervention? 2. Who- All potential participants (during recruitment) and then all participants consented to take part (during follow-up) receive it? 3. When: receives/delivers during the beginning of the trial (e.g. the informed consent process) and then throughout the trial (as a reminder of set goals) 4. Where: receives/delivers at trial site (e.g.NHS/clinic setting) or home (e.g. online trial or during reminder phase of expected tasks) 5. How often: receives/delivers during touch points between trial office and participants 6. How/MoD: receives/delivers as welcome pack (paper/electronic)/ Text message, Email, letter, telephone (e.g. reminder)/Newsletter   What would be the acceptability if (Intervention Motivational info):   1. Who: trial team/research nurse/a peer from the trial (e.g. online forum) deliver this intervention? 2. Who- All participants consented to take part will receive it? 3. When: discussed during recruitment consultation /’touch points’ between trial office and participants (e.g. newsletter/reminders) 4. Where: received/delivered at trial site (recruitment place e.g. hospital) or home (via email, text, mail, telephone) 5. How often: During touch points between trial office and participants 6. How/MoD: Delivers/receives as welcome pack (paper/electronic)/ Text message, Email, letter (e.g. reminder)/Newsletter   [What (other) format should this intervention take (mode of delivery)  -Where (home, trial office, hospital) (other) should this intervention take place?  -Any other suggestions?] |
| Perceived effectiveness: (anticipated/experienced): the extent to which the intervention is perceived to have achieved its intended purpose | 1. How much would you say the intervention can improve questionnaire return/clinic attendance? 2. Why do you think so? | -Would you say it is ‘unlikely’/’likely’ to influence retention? |
| Self-efficacy: the participant’s confidence that they can perform the behaviour(s) required to participate in the intervention | How confident do you feel about your role in delivering this intervention? (trial staff/RNs)  How confident do you feel that you could participate in this intervention? (participants/REC) | -Why/why not?  -What made you feel confident/less confident?  - Are there any specific skills and knowledge needed to participate/deliver this intervention? |
| Anticipated Opportunity costs: the extent to which benefits, profits or values must be given up engaging in the intervention | 1. Do you feel enabling the delivery of this intervention would interfere with other things you need to do? (trial staff/RNs)   Do you feel taking part in this intervention would interfere with other things you want to do? (participants/REC)   1. What additional resources would (does) you and/or your Unit need in order to deliver this intervention? (trial staff/RNs)   What support you think you might need from the trial office to help you use or engage with this intervention? (participants/REC) | -If yes, why do you feel so?  -Any other benefits and downsides to implement this intervention? (trial staff/RNs) |
| Other Qs | Is there anything else that I have not covered/you would like to discuss? |  |

**Table 3. Quotes from participants in the intervention co-production workshop.**

| **Intervention** | **Participant quotes** |
| --- | --- |
| **Incentives** | ‘…it can’t be a huge amount of money, it’s just recognition of the time that you’ve given to it and yeah, and it does take time to complete these studies, so that’s my own thoughts. I don’t know whether you could choose to opt for that money to go to charity’ P2_DISCO_Female  ‘When I first agreed to take part in it I didn’t know there was going to be vouchers, but when the vouchers did come it was, it was like £15 vouchers but I appreciated it, I thought it was useful. What I also felt that I was incentivised by the fact that I thought I was doing something useful by taking part in the study. For example, everybody wants you to do a survey these days don’t they, every time you go to Marks & Spencer or Tesco they want you to send back a survey. But I don’t do it now because I think, “Well whose benefit is this for? Is this for my benefit as a customer or is this for Marks & Spencer and Tesco’s benefit?” and, so I just don’t bother doing it now. But I could, I could actually see that taking part in this study hopefully would benefit me or other people so I thought it was useful………. And in terms of whether you like the vouchers or not, personally I thought it was covered and I thought the value of it was ok and it didn’t need to be something big, it was just like a thank you’ P1_INTERVAL_Male  ‘We felt we were also incentivised by it being useful therefore it’s financial and social. We felt if there is a financial incentive it shouldn’t be too much, bearing in mind it’s the NHS, so for example, £15, because we want people, the right people to participate not just for the financial gains.’ P5_MASTER_Male  ‘Also we said about vouchers might not be relevant to what you want. We mentioned -- oh, yeah, also online would be quicker and easier, maybe it could go straight into your bank account for instance, and we thought that a financial incentive was important because it’s kind of exponentially linked to how much time is required. So if you have to fill in a questionnaire and it takes you and hour and a half of your time to fill it in then actually that’s an hour and a half of your time that you’re not spending with your family, and if the trials going over for a significant period of time, over a year and you’re having to do this, I don’t know, once a month, then actually it’s quite a considerable amount of time. So a financial recognition is appropriate probably depending on the amount of time and contribution you might be making.’ P3_Various_Female  ‘And I think on the financial side, I mean the point you mentioned about actually is you know, £15 is great but actually if you’ve got to spend all day in the clinic to do a range of tests yeah, then actually £15 is like, well actually it’s a bit of an insult, I’d rather not get anything at all.’ P3_Various_female  ‘P7 - But wouldn’t, if you come clean at the beginning, wouldn’t people just say, “I’m going to get £15 out of this, I’ll do the first one and then drop out anyway”?  Researcher - So you’d make it a condition that they had to…  P4 - we can say this and we can say this…  P7 - But how do you take it back if you don’t complete it?  P3 - Yeah, but maybe you get £15 every time you complete it, or it’s exponential, so you get £10 initially and £15 for the second one…  P7 - Yeah, like a loyalty thing for every one you do as you go up, yeah, that will be a better way of doing it.’  ‘I wondered (inaudible - background conversation) something like a loyalty card, every time you returned something you get a stamp or something like that and it should, it should feel like (inaudible - background conversation) because nobody every said to me, “If you don’t complete this it’s going to screw up all of our data”. But they need everybody to do it for the full time don’t they, so there’s got to be a way of keeping you in it for the long haul.’ P6_INTERVAL_Female  ‘I’m not saying you shouldn’t have incentives, I’m just saying why does it have to be financial?....... Well I mean it’s like I said earlier though isn’t it, constantly reminded that the NHS is struggling, once you’ve taken the incentives out and money out of a struggling NHS when we need the treatment more than anything else you know, you spend enough on the treatment’ P7_MASTER_Male  ‘… or you get a really nice coffee voucher or a box chocolate’ P6_INTERVAL_Female  ‘But we felt the main incentive is, “I’m making a difference”’ P5_MASTER_Male |
| **Goal setting** | P1 - No, no, if they want us to set goals i.e. they want to say, “Well you need to do four questionnaires over a 12 month period and you will get this”, in return for that you should also get, “Well we promise to give you an outcome and keep you informed”.  P6 - Yeah, it should be about goals, the goal should be to get informed…  P7 - “You’ll get regular feedback and we’ll keep you informed of what’s happening”.  Researcher - So what do you think what goals should be set, it’s all questionnaires or do you think some of them, or how should (inaudible - background conversation)  P1 - No, I think if you sign up you sign up for the duration.  P5 - Yeah, I was quite surprised to be offered four out of six, I mean my perception is you do all of them.  ‘Yeah, it needs to be a personal approach and face to face for the format….. I think once you’ve been into the clinical trial and you get, I don’t know, a rapport with yourself, you sort of… if you get an email from somebody else you’re sort of sceptical about a trial. But if we got a text message from you saying, “We’re going to take this on the next stage”, we’d be expecting it, so again I put it it’s that personal approach again isn’t it.’ P7_MASTER_Male  ‘Goals to be set upfront or, and then the option to revise those during the trial. I know we were bit divided on that; some people like goal setting, some don’t, but we thought that maybe there was that option there that you could opt out if your life had changed or if life circumstances changed for you. Goal setting is there should be a clear timeline.’ P2_DISCO_FEMALE  P6 - But I’m trying to think of when we did the dental piece if someone had sat down with me and said, “What are the goals, can we agree some goals for the outcome of this?” I think for some people that might be a little bit off putting mightn’t it, goals, unless it’s it’s quite simple.  P5 - Yeah, simple goals.  P6 - And then the other side you think if you have the goals would you treat it more seriously rather than, “They don’t care if I return this or not”. Is there something about your commitments?  ‘Just going back to goals and to design, so if you’re setting a goal for, “Oh, I’m going to fill out all the questionnaires” or “I’m going to fill out three of the six questionnaires”, isn’t the whole point of the design that all the questionnaires are there for a reason, so we shouldn’t really set a goal because if you miss them out then actually it has an impact on the trial? ‘ P3_Various_Female |
| Participant self-monitoring | P7 - Perhaps you ought to send out a little diary…  P5 - Yeah, a free diary.  P7 - … but make sure it’s got your research logo on it.  P6 - Got to be at home, you’ve got to have it at home all these reminder things.  P6 - You’ve got to do self-monitoring with the goal setting haven’t you; they’ve got to be combined.  R3 - That’s what we’re thinking, we can  P6 - It’s got to be combined because you’ve got to say, “Well if you’re going to commit to six out of six this is what they’re going to look like and then we’re going to give you this diary or this fridge magnet to remind…”  ‘Well I suppose you could end up with a bit of a multi-communication approach couldn’t you, because you get people who might opt in to receive information by email, and that’s very easy then when the information comes back you just ping them out an email saying, “Thank you, got it. By the way, you’ve done four or five, keep going, there’s two more to come”, and then you get your sticker, and that’s back to more of the continuous engagement thing. So you always feel like there’s a touch point every so often.’ P6_interval_Female  ‘We mentioned calendars, we mentioned Outlook. Well expiry date of questionnaires to avoid procrastination, definite dates and in fact that’s probably something that perhaps should be agreed before anything takes place as to when the results are… should be returned.’ P8_INTERVAL_Male  ‘I think another thing we looked at is if there was going to be a physical, like a wall planner sent out or calendar, that the researchers probably know upfront when they want stuff returned by, that could automatically put (overspeaking) so this two week period. For me, I prefer to upload something to the cloud, so if I had virtual calendar I could upload it to the cloud for the results and what have you. But we looked at you know, especially around the deadlines, if there was something physical that people could… I say physical, either virtually on the cloud or in the actual planner, see on that when stuff’s got to be returned by. ‘ P2_DISCO_Female  ‘Yeah, the number of times all the kids stuff come through and it’s like you put it on the fridge and you forget about it and then all of a sudden one day you’re just passing and I think, “Oh, my god, that’s tomorrow”, you take it down and fill it in or, yeah. So I think that’s maybe also why you mentioned about the fridge magnet like this. I know not everyone has a magnet fridge, but to me it’s like it’s actually really quite practical.’ P3_Various_Female  ‘I think in terms of the format, I mean clearly there are several of us who are quite technologically versed and are really happy with doing stuff online. There may be other people who that’s not the case for and they’re still happy with paper based questionnaires, and we’re not quite sure where you go in between those.’ P1_INTERVAL_Male  ‘So we thought that you could include things like loyalty card, fridge magnet, a diary or a calendar. And then we also thought that it’d be quite nice if you got on your loyalty card after every either clinic visit or questionnaire submission you got a little gold star or something like that and you could apply it to your little card.’ P6_INTERVAL_Female |
| Motivational information | P6 - I think there are a lot of parallels with charities in this…  P1 - Do you?  P6 - A lot of parallels the way charities talk to you about contribution…  P5 - Yeah, your contribution.  P6 - … like “You’re making a difference, your contribution makes a difference”.  P5 - Yes, I don’t like it coming across as a threat.  P6 - Carrot or stick.  P5 - Yeah.  P1 - Tell people why what they’re doing is a good thing to do.  ‘And I would say throughout the study keep reinforcing the message otherwise people… I mean if it kept informed throughout… if it goes on over an extended period of time so people aren’t thinking, “Well why am I filling in another one of these questionnaires in?” just keep telling them what the idea of it is and what it’s hoping to achieve. Keep thanking people for contributing.’ P1_INTERVAL_Male  P7 - I’m guessing it needs really to be sort of telephone approach so that you keep that personal touch.  P1 - Yeah, keep the personal touch.  P1 - So there is a theme running through all this stuff of trying to find out what works best for each individual participant…  P6 - Yeah, it’s the tailoring again.  ‘We mentioned around being upfront, a lot of things around communication that we’ve touched on before and having a point of contact. Also encouragement messages, especially part of the way through when people might start flagging. We mentioned about peer support, a bit like Weight Watchers and methods to do that; that might need moderation or facilitation as well. Let me see, other things. Aspirational messages, so like maybe case studies, so part of the way through you might want a case study to say, “In another trial that was of a similar profile this is what happened and these…” and say like “The guidelines were changed and clinical practice changed because of a trial” and that’s like the end goal. So I think a lot of us also mentioned that a lot of what you’re trying to do is for the greater good or impact of further generations, so actually it’s like a really aspirational message to see what the end goal is’. P3_Various_Female  ‘In addition to that saying is there anyone you can discuss it with? It’s all very well writing to someone saying, “We noted you haven’t sent back”, but it may be that you have a question about it as to why they haven’t. And I think we’ve been discussing all the way through that this point of contact with whoever’s involved in the study, even just have a word with them, perhaps support (inaudible) “Yes, you’re doing very well in the study” or if there’s anything you’re not understanding.’ P4_INTERVAL_female  ‘That would motivate me definitely or the next day, that would motivate me knowing that I’m perhaps putting people at risk in the future.’ P8_MASTER_Male |

**Table 4. Quotes from trial stakeholders in focus groups exploring intervention acceptability.**

| **Goal Setting Intervention** |
| --- |
| ‘So I suppose the goal setting for retention makes me a bit anxious at the time of trial entry because there’s an awful lot of information that people need to take in, and it depends on the situation doesn’t it, if you’re considering surgery versus not surgery, that’s probably what most people are thinking about at the time, not thinking whether they’re going to be able to fill in a questionnaire nine months later, and that will dominate their thoughts. And in my… the trials that I run, which almost always recruit in labour, that’s obviously not going to be what is at the top of most women’s minds when they’re in labour. So I think it’s right that one should do that, but I don’t think we can expect much from that at that time point. So to me this I think is mostly about what we could do after they’ve actually… people have actually entered the trial and then planned their… you know, once that is all… because it’s such a big thing to take part in a trial, there’s so much information that this probably gets… it comes much further down the line doesn’t it I would suspect,’ Trialist_01  ‘I totally agree that there’s got to be transparency upfront, that “This is the expectation, these are the time points”, this is the expectation, I would agree that the perhaps the goal setting needs to take part a little bit after that once they’ve got… it very much depends on the type of trial. So what we’re talking about I think one size if not going to fit all as is the same with all trials, so it’s very much dependent on the trial. But from a trial manager’s perspective, I think talking to my… the research nurses about that recruitment consultation, sometimes that’s a very busy clinic you know, they’ve been pulled out of something else, or a clinician to get this patient consulted, I’m not for a minute saying that they would rush a consultation, I just don’t think that perhaps in some studies that goal setting would sit naturally in that recruitment consultation.’ Trial Manager_01  ‘I agree with that actually because if you’re not told, and it sounds(?) like the [trial name] one was a little bit vague to start with, then it might be one of the reasons that people sort of think, “Oh, not another questionnaire, I didn’t realise there was going to be this amount”, and they just lose interest at that stage as well. I do definitely think that the more transparent, the key facts particularly are important and they could be initially in the conversation as well, the patient has then got the information to go up and have a look in more detail with the pack(?) that they’re given.’ Trial Participant_01  ‘I think one of the reasons people are having trouble talking about goal setting precisely is because you’re not really talking about goal setting are you, you’re not saying, “Let’s agree a goal with the patient”, you’re saying, “Let’s find more ways of persuading the patient to do what we want”. Now when I think of goal setting I think of you know, “How many questionnaires do you think you could fill in out of…” let’s say a lot like ten, “Well I could probably do seven”, “Well you know, maybe we could help you to do eight”. Whereas you’re saying you know, all the examples you’ve been giving are, “You want you to fill in six, but if we ask you how many you think you can fill in and however many you say we’re going to try and persuade you to do six”. So it’s not really goal setting, it’s just persuasion. And, so the bit that might be different, it might be okay, so we’ve managed to highlight the fact that this person already thinks they’re going to find it hard to fill in that number, therefore we should look at ways of motivating them to do it, but that’s not the same as goal setting.’ REC member_01  *Trial Manager_02* ‘So just following up on that, if somebody had said to you in that way, so you’ve done your informed consent, you’ve had it all kind of… all the information’s down(?) and then a little while later you, three days or whatever, you see somebody and they say, “Okay [name], how many of these questionnaires do you think you’re going to be able to fill in over the next…” however long it was, what would you have said?  *Trial Participant_01* ‘My initial response would be, “I’m obviously part of it so how many do you want me to fill in?” and if you said 12 or if you said 20 you know, everybody will say, “Of course I’ll try and do the 20”, because you feel then as though you’re trying to make a difference.’  *Trial Manager_02* ‘And if somebody said, “Well could you write down, ‘Yes, I agree that my goal is to fill them all in’?” would you do that and would it make any difference?  *Trial Participant_01* - I think I would fill in saying, “I will do my best to fill 20 in”, I wouldn’t commit myself to doing 20 because with all the best will in the world nobody can commit themselves for two years. But if you come into these trials you know that you’re agreeing to do X, Y and Z, and I think once you’ve agreed to do it you should stick to it and you should do it, make that…’  ‘So I had took goal setting to be getting everybody to do what they’ve signed up to do, which is effectively if we’re open and transparent at the beginning and you know what the trial is then people are going into it expecting to do that. And there’s something more than that, which is a way of trying to help them deliver what they had initially said to do.’ Trialist_01  ‘I think that’s right, I think what we’re talking about in terms of goal setting, and the terminology that seems very, very formal and a little sheet saying, “I will commit to completing this many questionnaires”. But actually in reality, and (inaudible) will probably have a better input into this, is it more about just having that conversation saying, “How many questionnaires do you think you’d be able to manage?” not even having that conversation, just saying, “We would like as many as possible back. Obviously there are going to barriers to this, what do you think those barriers might be and how can we help you?” Trial Manager_01  ‘Yeah, it’s just some of this just seems a bit common sense. I can’t see why for example, well this isn’t a burden on the patient, the participant, but I can’t see why you would want poster information and newsletters if you’ve got people’s emails and text and they’ve agreed to get in touch with you. I can’t see why you would want… I mean we would be very anxious about any kind of contract or anything on an informed consent form that said, “I promise to do this many”. And I think the idea of sitting down and setting a goal and forming an action plan I would just think feels slightly unnecessary bureaucracy, whereas something that said… discussing how people would like to be communicated with and giving them an example of the questionnaire they might need to fill in would make perfectly good sense.’ REC Member_01  ‘Yeah, so with my experience we’ve got frail older people, so I think they’ve got loads of burdens that goes through with participating in trials. But what I found out is with my experience, if it’s not patient centred it wouldn’t work out. So when we start, that’s why I said from early on we try to take things slowly and be with the patient from the beginning to the end, and building that relationship, as what you’ve said, we’re already started doing… “We’ve got some questionnaires, when do you think shall we come to do have this… to work it out with you?” and stuff like that. So I think for me with the trials that we’re doing I think it is not a burden, it will actually help us to put the patient at the centre and what would they like to do, that’s…’ Research Nurse_01  ‘I also think there’s a massive burden at the minute on research nurses, again and it varies so much from trust to trust in terms of some research nurses have got so many trials, you walk into an office and it’s wall to wall folders with all the different trials. So from our perspective if you like it’s an extra sort of quarter of an hour, half an hour to have a conversation with this participant about goal setting (several inaudible words) so actually that’s not a big deal, but to the research nurse’s point, “I manage 30 trials already”, actually an extra hour has got them thinking, “Oh, my word…” And it will be trying to get across the value of it I think’ Research Nurse_02  ‘I’m thinking as a trial manager this sounds great and anything that we can do to hopefully improve our responses is fantastic, but how we put this into practice, because that consultation is already… there’s a lot of information to get through to discuss during the consent and the information that you’re sort of sharing and getting, and to add something else into that when there’s already so much going on, is that the best… I know you can talk about these things, but given that we said as well that sometimes the information’s not being shared correctly, is it the best… when do we do it in a time that’s already really busy, and then do we need to follow that up with something additional directly after the consultation when somebody is in the trial to remind them about all of the things that we’ve chatted about?’ Trial Manager_03  ‘I think the other thing as well is when you say, “You’re going to get three questionnaires” the nurse who doesn’t say that begins, “You’re going to get a questionnaire but if you don’t return it you’re going to get another and then another”, and that’s when people start getting confused and start really worrying, “You’re sending me all these questionnaires, what do I do?” and then they start pulling out because they’re not … they’ve disengaged at that point.’ Trialist_02  ‘So if there’s maybe something that we’re missing that we could be doing better in that document (patient information leaflet] that would further support things as we go along the process, and is there a clearer section in there that we could put in about on to this goal setting you know, are all patient information leaflets being very clear about what the follow up period is and what the expectation is within that?’ Trial Manager_01  ‘You could have that discussion where you’re sort of setting those goal type behaviours, so at that point you might say, “We appreciate that life is busy and sometimes it isn’t possible to complete all of the questionnaires, but we want to highlight these are the number of the questionnaires that you could expect”. I don’t know, I just feel it opens up a discussion that might not be happening even with the best will in the world that maybe isn’t happening at the moment, that may be some way of trying to get that discussion in at the point they’re consenting and coming in and they should understand everything that’s going to happen, it’s there, they go home to a copy of the consent form and it’s still there. I feel it’s part of their fully informed decision.’ Research Nurse_03 |
| **Motivational Information Intervention** |
| ‘I can’t see any point. If I was a participant, I’d much rather hear from the clinical team than from some other random people who happen to be in the same trial.’ REC Member_01  ‘The one, both, probably more for a, from the ethics committee side of me, to remind them of the benefit of being involved, to me would become more as a coercion, that they’re…you know. That they’re being coerced into continuing to be involved ‘ REC Member_02  ‘And the other thing was the aspirational messages, I think as a research nurse, feel like I was, you know, preaching. That’s how that reads to me, that I’m pushing it, that I’m encouraging people by giving them an aspirational message. I think it’s just the wording.’ Research Nurse_02  ‘And I think the temptation there would be to put something that changes clinical practice in a positive way, and of course if the trial then doesn’t demonstrate that or, even worse case, causes harm, you’ve almost put a motivational message in there which is that by taking part in this you will change the code of practice in a good way, so it does have to be handled quite carefully that. How you provide that message. But the principal of, because I think the general public in general don’t know what clinical research, what clinical trials contribute to the way we provide NHS care. And that’s because they’re never told, we don’t’ explain that very clearly. So, that is good. But that’s often in the patient information at the beginning, isn’t it, the patient information. Because we often talk about the context that this is happening.’ Trialist_01  ‘we use a lot of the motivational information anyway in reminder letters,…. You need to be a wee bit careful about…there’s so many time points and if you’re sending the same letter at each time point, you’ve got to be careful that you’re not just lessening the message by becoming quite repetitive. Like, you need to find different ways to say the same thing, at all these different time points. And I think that would be, that’s the headache in terms of implementing it, but I think if you say the same thing every single time, then you stop reading it. because it’s the same letter’ Trial Manager_02  ‘And they found that they got a lot more people sending their tax returns back when they said, rather than saying, ‘you haven’t sent it in, send it in’, they said, ‘most people do send their tax return in time’ and then that made people do it. So, I think there would be ways of phrasing it that would be likely to work and say, most people send them back on time, but we do really need 100% back on time if we’re going to get the best possible results, or something like that.’ Trial Particiapnt_03  ‘So, all the motivations we‘ve got so far are quite general aren’t they, they’re aimed at everybody. But we haven’t got anything which is more personalised to the individual. And, you know, I’m conscious that we should be able to with our, in our new electronic age, be able to do that a bit more. Where we can target people. So, I suppose it comes back to the previous discussion we had, and if you’ve got eight questionnaires you want filled out over a year, you could almost, we should be able to sit the systems out so we almost return an individual score card if you like, to the individual, saying, ‘it’s coming up to that time again, you should have a questionnaire, thank you for filling all the questions out so far’, or, ‘I know you’ve missed one, we’d really like your questionnaire this time around’. I mean, if it’s possible, those individual motivating things would probably be even more effective, is my suspicion. Rather than general motivation. Something which is absolutely aimed at the person.’ Trialist_01  ‘I think it would be less work for participants, because obviously they’re responding to what’s required. I personally, again, it’s preaching to the converted, I’ve signed up to this because I want to be able to contribute, so if there are people who are thinking, ‘I’ve had enough of this’, then obviously the work involved in encouraging them to have the motivation is important, but it’s working out at about 10% increased retention, I don’t know, because I’m not entirely sure what work would be involved in trying different methods, I know there’s more motivation(?).’ Trial Participant_02  ‘I have one quite small comment on some of the examples, I’ve got one example here which is keep thanking people for their contribution. I think thanking is very easy to lose its power and impact and being sincere, so keep thanking immediately makes me thing that’s got to be insincere if you know you’ve just got to keep thanking people. So, I think thank people proportionately and sincerely and at the right moment. Like, when they agree to take part and when they’ve finished. But if every time you send a questionnaire and you get an email saying, ‘oh thanks for filling the questionnaire’, it’s kind of, well blimming hell.’ Research Nurse_03  ‘Personally, for the majority it would be fairly straight forward. Even to implement a lot of the things that we’re doing already.’ Trial Manager_04  ‘So, the peer support just needs a bit of thinking about. I’ve done it for one trial, quite a long time ago. It had to be moderated, because people who had a bad outcome, there was one couple who had a bad outcome who got very upset and took it out on the trial and their consultants, and they were devilling people on the social medias, so one has to be a little bit careful about that. I have to say, I haven’t tended to do it since, I’ve often thought it would be a good idea, but I don’t know what others feel about what experience they’ve got? I quite like the concept of, that people in the trial are communicating with each other.’ Trialist_01 |
